# Evolutionary analysis of KED-rich proteins in plants

**DOI:** 10.1371/journal.pone.0279772

**Published:** 2023-03-08

**Authors:** Xing-Hai Zhang, David Swait, Xiao-Lu Jin, Paveena Vichyavichien, Nicholas Nifakos, Noah Kaplan, Lucwilerna Raymond, John M. Harlin

**Affiliations:** 1 Department of Biological Sciences, Florida Atlantic University, Boca Raton, Florida, United States of America; 2 Penta 5, USA, Sarasota, Florida, United States of America; SOKENDAI (The Graduate University for Advanced Studies), JAPAN

## Abstract

During the course of evolution, organisms have developed genetic mechanisms in response to various environmental stresses including wounding from mechanical damage or herbivory-caused injury. A previous study of wounding response in the plant tobacco identified a unique wound-induced gene, aptly named *KED* due to its coding for a protein that has an unusually high content of amino acids lysine (K), glutamic acid (E) and aspartic acid (D). However, by far little is known about this intriguing gene. In this study, we investigated the evolutionary aspects of the KED-rich coding genes. We found that a consistent pattern of wound-induced *KED* gene expression is maintained across representative species of angiosperm and gymnosperm. *KED* genes can be identified in species from all groups of land plants (Embryophyta). All the KED proteins from vascular plants (Tracheophyta) including angiosperm, gymnosperm, fern and lycophyte share a conserved 19-amino acid domain near the C-terminus, whereas bryophytes (moss, liverwort and hornwort) possess KED-rich, multi-direct-repeat sequences that are distinct from the vascular plant KEDs. We detected KED-rich sequences in Charophyta species but not in Chlorophyta wherever genome sequences are available. Our studies suggest diverse and complex evolution pathways for land plant *KED* genes. Vascular plant KEDs exhibit high evolutionary conservation, implicating their shared function in response to wounding stress. The extraordinary enrichment of amino acids K, E and D in these groups of distinct and widely distributed proteins may reflect the structural and functional requirement for these three residues during some 600 million years of land plant evolution.

## Introduction

The green plants (Viridiplantae) are thought to have originated on Earth some 900 million years ago (mya). They evolved into diverse groups of Chlorophyta, Charophyta and Embryophyta (land plants). The land plants are believed to have begun inhabiting on land some 600 mya. About 450 mya, vascular plants (Tracheophyta) emerged from the land plants, followed by appearance of seed plants (Spermatophytes) some 320 mya [1–3]. Throughout those hundreds of million years of evolution, all living organisms have evolved various genetically directed mechanisms to respond to and deal with constantly changing environmental conditions and challenges. For example, plants unavoidably experience various stresses such as wounding by mechanical damage or animal attacks. In response, plants have developed sophisticated mechanical stress or wound-response network involving hundreds of wound-responsive genes [4, 5]. In a screening of wound-responsive genes in tobacco, Hara et al (2000) [6] identified a gene coding for a unique protein that is unusually rich in lysine (K, 34.7% of total amino acid composition), glutamic acid (E, 25.0%) and aspartic acid (D, 12.5%), therefore named KED. Transcription of this *KED* gene was induced by wounding as soon as within 10 minutes, elevated to a peak within 1 h and decreased to baseline after 2 h [6]. However, its function or biochemical activity remains speculative. To our knowledge, there is no other published study of this *KED* gene in literature since and there is no *KED* mutant reported.

KED protein’s unusual residue composition and its rapid and transient early induction of transcription in response to wounding may hint at its niche biological function, as the doctrine “structure dictates function” implies. We are intrigued by questions such as: are *KED* genes widely present in plants and other species? How conserved is this protein across taxonomical groups? Is there any evolutionary or functional implication for a protein with such huge bias for K, E and D over other available 17 amino acids? As part of our efforts to understand the function of this enigmatic protein and more broadly plants’ stress response, we report here our examination of evolutionary aspects of genes coding for KED-rich proteins. Our studies reveal a wide presence of the *KED* genes among land plants (Embryophyta), implicating its evolutionarily conserved function in plant life. Its marked conservation among vascular plants (Tracheophyta) may suggest evolutionary selection and functional requirement for enrichment of K, E and D residues in the DNA coding sequences as part of plant response system to wounding or mechanical stresses.

## Materials and methods

### Plants and wound treatment

Seeds of tomato (*Solanum lycopersicum*, cv Micro Tom), *Arabidopsis thaliana* and corn (*Zea mays*) were germinated and grown in soil pots. Plants (height 20–25 cm) of white spruce (*Picea glauca*) were purchased from Nature Hills (USA). These plants were grown in a growth room at 25°C under 8 h of darkness and 16 h of cool white light of 250 μmol photons m^-2^ s^-1^. Tubers of waterlily (*Nymphaea colorata*), an aquatic angiosperm, were purchased from Greenpro (USA) and grown in the field near the university campus in south Florida.

### Expression analysis of *KED* genes

Unwounded leaves were collected immediately before wounding treatment and stored at -80°C. For wounding treatment, leaves from the same plants were pinched with a pair of tweezers as described by [7]. After 0.5 h and 1 h, the wounded leaves were collected sequentially. Leaves (unwounded, and wounded for 0.5 h and 1 h, all from the same plants) were ground in liquid N_2_ to fine powders with mortars and pestles. RNA was extracted using Total RNA Mini kit (plant) (Geneaid/FroggaBio, USA) with DNase treatment. First-strand cDNA was made using SuperScript IV reverse transcriptase (Invitrogen, USA) and a mixture of oligo dT and random primers according to the manufacturer’s instruction.

Quantitative real-time PCR (qPCR) using SYBR Green (Life Technologies, USA) was carried out to measure the relative *KED* transcription levels in leaves of tomato, Arabidopsis, corn, spruce and waterlily. Reference genes for ΔCt normalization were tomato phosphoglycerate kinase [8], *Arabidopsis thaliana TIP41-like* protein [9], corn *Elongation factor 1-α* [10], white spruce *Elongation factor 1-α* [11] and waterlily *actin* [12]. At least three plants from each species were tested. The primer sequences are listed in S1 Table in S1 File.

### Genomic database searching and sequence retrieving

The nucleotide and amino acid sequences for tobacco (*Nicotiana tabacum*) KED [6] were used as a starting point for the initial search using BLASTN and BLASTP tools against the GenBank and OneKP database including non-redundant nucleotide and protein sequences, whole-genome shot gun, expressed sequence tags, high throughput genomic sequences, UniProtKB, transcriptome shotgun assembly proteins and protein data bank. Initial search using both the coding nucleotide sequences and the amino acid sequences identified 32 eudicots and at least one monocot (*Elaeis guineensis)*. Subsequent searches were performed against the *E*. *guineensis* amino acid sequence through Liliopsida (monocotyledons) database to identify matching sequences of monocots. Likewise, using retrieved sequences to systematically search databases of the same orders and families of eudicotyledons yielded more species of possible matching sequences. Similar strategy was used to identified KED sequences from gymnosperms. Further searches were done sequentially by narrowing organism groups to find matches from more closely related species. However, it must be pointed out that the database search was aimed at surveying broadly the possible taxonomic presence of the *KED* gene and the retrieved sequences are not by no means an exhaustive outcome due to genomic sequence availability and the annotation quality of the public databases.

To search for possible KED-rich sequences in charophytes, bacteria and animals, KED protein and nucleotide sequences from plants were first repeatedly blasted through each of the intended organism groups in the databases. Then each match was further examined by retrieving its sequence from the database. Translation tool was used to generate open reading frames, followed by amino acid composition analysis, specifically for K+E+D content, to score the putative KED candidates. Once a KED sequence was identified from one taxon group (for example, charophyte), this sequence was used to search the entire available entries from this group. This way, sequences predicting KED-rich open reading frames in genomes of several charophyte, bacterial and animal species were identified.

During the course of searching animal KED candidates, a 6,229-amino acid microtubule-associated protein futsch from honeybee (*Apis cerana*) was found to contain an internal KED-rich region, whereas its N- and C-terminus portions have normal K, E and D contents. To illustrate examples of the presence of KED sequences in animal species, this 750-amino acid internal KED-rich region was arbitrarily taken out for demonstration in this study.

All retrieved sequences of possible matches were manually reviewed and verified for proper open reading frames and translated sequences. Wherever applicable, both genomic sequences and mRNA sequences were matched to verify the correct coding sequences. The full-length, translated sequences with considerable sequence identity and a high percentage of KED (K+E+D% greater than 30%) were designated as a candidate match.

Only partial KED sequences were available for two plants: cedar (*Cryptomeria japonica*, a gymnosperm; without C-terminus) and barley (*Hordeum vulgare*, a monocot, angiosperm; without N-terminus). However, they both still possessed the conserved domain (see “Results” below), therefore were included in sequence comparison analysis. But because their KED protein lengths were unknown and would distort the analysis parameters, they were excluded from the dataset for phylogenetic analysis described below.

### Phylogenetic and sequence analyses

Both the nucleotide and amino acid sequences were used to phylogeny analysis. All sequence files were first loaded into MEGA-X software (Molecular Evolutionary Genetics Analysis). MEGA-X Version 10.1.8 was used to align each set of sequences. MUSCLE alignment was performed on each sequence set with a -400.00 gap open penalty (default), 100 max iterations, and cluster method–UPGMA (default). Then a phylogeny was constructed in MEGA-X for each set using the maximum likelihood statistical method with 5000 number of bootstrap replications, all other options were set to their default settings. Afterwards, a “.nexus” file was exported out of MEGA-X with the MUSCLE alignment for each sequence set and loaded into MrBayes version 3.2.7a-win64. mcmcp ngen = 1,000,000 setting was used to set number of generations to a million. Lset nst = 6 setting was used to set the likelihood model parameters to allow all rates to be different, subject to the constraint of time-reversibility. All other settings were kept default. Different phylogenetic trees were constructed with either MEGA or BEAST (Bayesian Evolutionary Analysis Sampling Trees) platforms, which showed similar species positions. Because the BEAST method requires for time calibration priors, only MEGA trees are shown in “S1 File”.

Consensus sequences of the conserved domain were generated using EMBOSS CONS (www.ebi.ac.uk/Tools/msa/emboss_cons/). Pictograms of the consensus sequences were produced by using Weblogo (weblogo.Berkeley.edu).

### Residue abundance analysis

Each of 20 amino acids in a protein can be encoded by 1 to 6 codons among a total of 61 sense codons. Each codon is made of 3 nucleotides. Therefore, the abundance of each amino acid in a protein is overall pertaining to the abundance of its codon(s) as a result of evolution and variation of the nucleotide sequence making up all these 61 codons. However, codon bias is widely present across diverse species [13], which would contribute to considerable variations from codon-based predictions. On the other hand, amino acid relative abundance is also affected by both metabolic (production) cost and amino acid decay rates [14]. For this study, the expected percentage of an amino acid in a protein was assessed by both the genetic code model and the proteome analysis [14]. The actual percentage of a residue in the protein is the number of this residue divided by total residue (amino acid) number of this protein. The degree of discrepancy between the expected abundance and the actual abundance indicates the bias or enrichment of a particular residue in a protein by evolution.

## Results

### *KED* gene expression is wound-induced in angiosperm and gymnosperm plants

**Fig 1:**
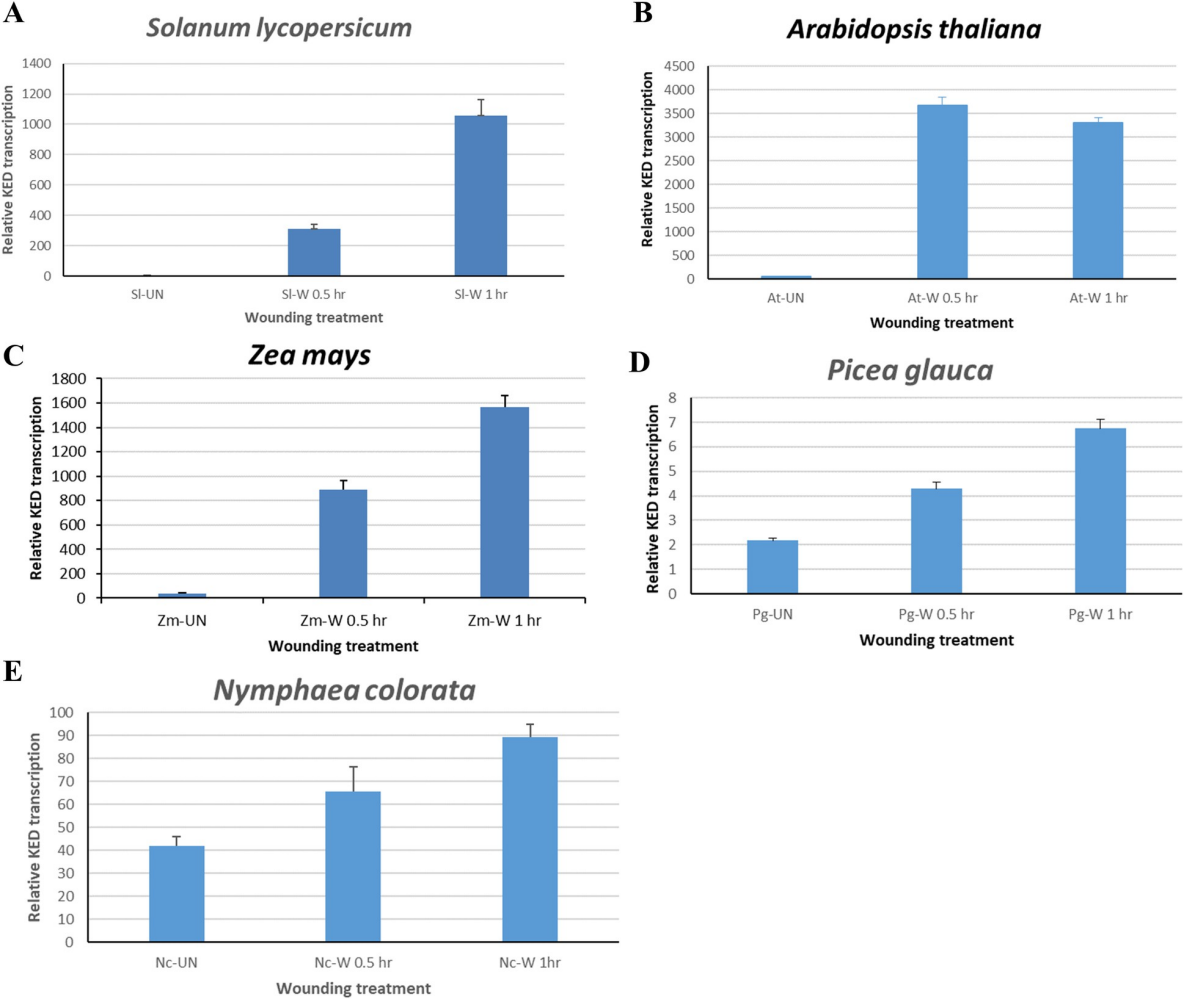
Wound-responsive transcription of *KED* genes in representative plants. **A.** *Solanum lycopersicum* (tomato; eudicot, angiosperm); **B.** *Arabidopsis thaliana* (eudicot, angiosperm); **C.** *Zea mays* (corn; monocot, angiosperm); **D.** *Picea glauca* (spruce; conifer, gymnosperm); **E.** *Nymphaea colorata* (waterlily; aquatic angiosperm). UN: un-wounded; W: wounded for 0.5 hour or 1 hour. Error bars denote standard deviations.

Sequences similar to the tobacco *KED* were identified from many other plants. Using gene specific primers, RT-qPCR was carried out to examine transcription pattern of five representative species in response to wounding. As shown in Fig 1, *KED* transcription was rapidly induced by wounding treatment in all these plants including eudicots (angiosperm) tomato and Arabidopsis, monocot (angiosperm) corn, conifer (gymnosperm) white spruce, and aquatic, basal angiosperm waterlily. This expression pattern is the same to that reported in tobacco [6], suggesting that KED’s wound-inducible transcription pattern is likely conserved in species across the seed plants (spermatophytes) angiosperm and gymnosperm. This prompted us to further investigate its taxonomic distribution and evolution.

### *KED* gene is widely present in vascular plants (Tracheophyta)

Extensive searching of available databases resulted in identification of KED-coding genes in 98 species of vascular plants, representing all major groups of Tracheophyta: spermatophytes, ferns and lycophytes. The seed plants (spermatophytes) include 68 eudicots, 19 monocots, along with 3 basal angiosperm species (*Amborella trichopoda*, *Nymphaea colorata* and *Nymphaea thermarum*) and 3 gymnosperms (Table 1, S2 Table in S1 File). They encompass 16 Orders and 27 Families of the eudicots, 5 Orders and 7 Families of the monocots, and 2 Orders and 2 Families of the gymnosperms (Table 2). [K+E+D] residue content of the predicted proteins from these 93 species ranges in 48.9% to 74.9% (Table 1). The predicted protein size varies from 224 to 673 amino acids (S2 Table in S1 File).

**Table 1:** Summary of calculated parameters of land plant (Embryophyte) KED-rich proteins.

Clade		Species	K%	E%	D%	KED%
**Tracheophyta (vascular plants)**	**Spermatophytes (seed plants)**	**Angiosperm: Eudicots (68 species)**	27.4 ~ 37.3	17.0 ~ 28.5	6.0 ~ 16.3	51.4 ~ 74.9
		**Angiosperm: Monocots (19 species)**	25.4 ~ 33.9	11.7 ~ 25.6	5.6 ~ 14.4	48.9 ~ 67.6
		**Angiosperm: Basal** *Amborella trichopoda*	34.4	24.4	11.4	70.2
		**Angiosperm: Basal** 2 *Nymphaea* species	26.3 ~ 26.5	20.4 ~ 21.4	6.3	70.2
		**Gymnosperm: Conifers (2 *Picea* species)**	28.7 ~ 29.2	14.2 ~ 14.6	12.5 ~ 12.9	55.8 ~ 56.8
		Gymnosperm: Conifer (one *Cryptomeria* species*)	34.8	16.3	20.2	71.3
		**Fern: *Adiantum capillus-veneris***	26.7	19.3	11.1	57.1
		**Lycophyte: *Selaginella moellendorffii***	35.1	22.4	11.5	69
**Bryophytes**	**Setaphyta**	**Moss: *Physcomitrium patens***	35.5	25.4	8.9	69.8
		**Moss: *Ceratodon purpureus***	32.6	26.4	8.9	67.9
		**Liverwort: *Marchantia polymorpha* (2 isoforms)**	30.1	23.6	19.3	73
			40.7	29.7	8.7	79.1
		**Hornwort: *Anthoceros angustus***	31.8	32.5	7.1	71.4

* Partial sequence truncated at C-terminus

**Table 2:** Taxonomic presence of seed plant (Spermatophyte) KED proteins.

Eudicots (68 species)			Monocots (19 species)			Gymnosperms (3 species)	
Order	Family	Order		Family	Order		Family
Rosales	Rosaceae (8)	Poales		Poaceae (11)	Pinales		Pinaceae (2)
	Rhamnaceae (1)			Bromeliaceae (1)	Cupressales		Cupressaceae (1)
	Cannabaceae (1)	Asparagales		Orchidaceae (2)			
	Moraceae (1)			Asparagaceae (1)			
Malpighiales	Euphorbiaceae (3)	Arecales		Arecaceae (2)			
	Salicaceae (2)	Zingiberales		Musaceae (1)			
Fabales	Fabaceae (15)	Alismatales		Araceae (1)			
	Fagaceae (2)						
Cucurbitales	Cucurbitaceae (2)						
Myrtales	Myrtaceae (3)						
	Lythraceae (1)						
Malvales	Malvaceae (5)						
Brassicales	Caricaceae (1)						
	Brassicaceae (5)						
Sapindales	Anacardiaceae (1)						
	Rutaceae (1)						
Vitales	Vitaceae (1)						
Gentianales	Rubiaceae (2)						
Lamiales	Oleaceae (1)						
	Phrymaceae (1)						
	Pedaliaceae (1)						
Solanales	Solanaceae (5)						
	Convolvulaceae (1)						
Apiales	Apiaceae (1)						
Ericales	Theaceae (1)						
Proteales	Nelumbonaceae (1)						
Ranunculales	Papaveraceae (1)						

Note: Number in parenthesis indicates species numbers. Angiosperm basal species *Amborella trichopoda* and two *Nymphaea* species are not included.

It may be worth pointing out that besides terrestrial (land) vascular plants, the *KED* gene was also found in two groups of aquatic vascular plants: the eudicot lotus *Nelumbo nucifera* and the angiosperm waterlilies (*Nymphaea colorata* and *N*. *thermarum*) (Table 1, S2 Table in S1 File). Waterlilies are considered as the second branching of angiosperms, after the first basal branch *Amborella* [15]. However, *KED* gene was not detected in the genome of *Ginkgo biloba* [16], a so-called “living fossil” thought to be existing for 290 million years.

Phylogenetic trees constructed using the KED amino acid or nucleotide sequences from the seed plants (spermatophytes) are consistent to each other overall (S1 and S2 Figs in S1 File), which are generally consistent with the well-accepted taxonomic relationship. This suggests congruence between KED gene variation and species evolution (Table 2).

DNA sequences coding for KED-rich proteins were also found in one fern species (*Adiantum capillus-veneris*) and one lycophyte species (*Selaginella moellendorffii*) (Table 1, S2 Table in S1 File), which complete representation of the monophyletic group of vascular plants (Tracheophyta) within land plants (Embryophyta).

### All vascular plant KED proteins examined share a conserved domain

**Fig 2:**
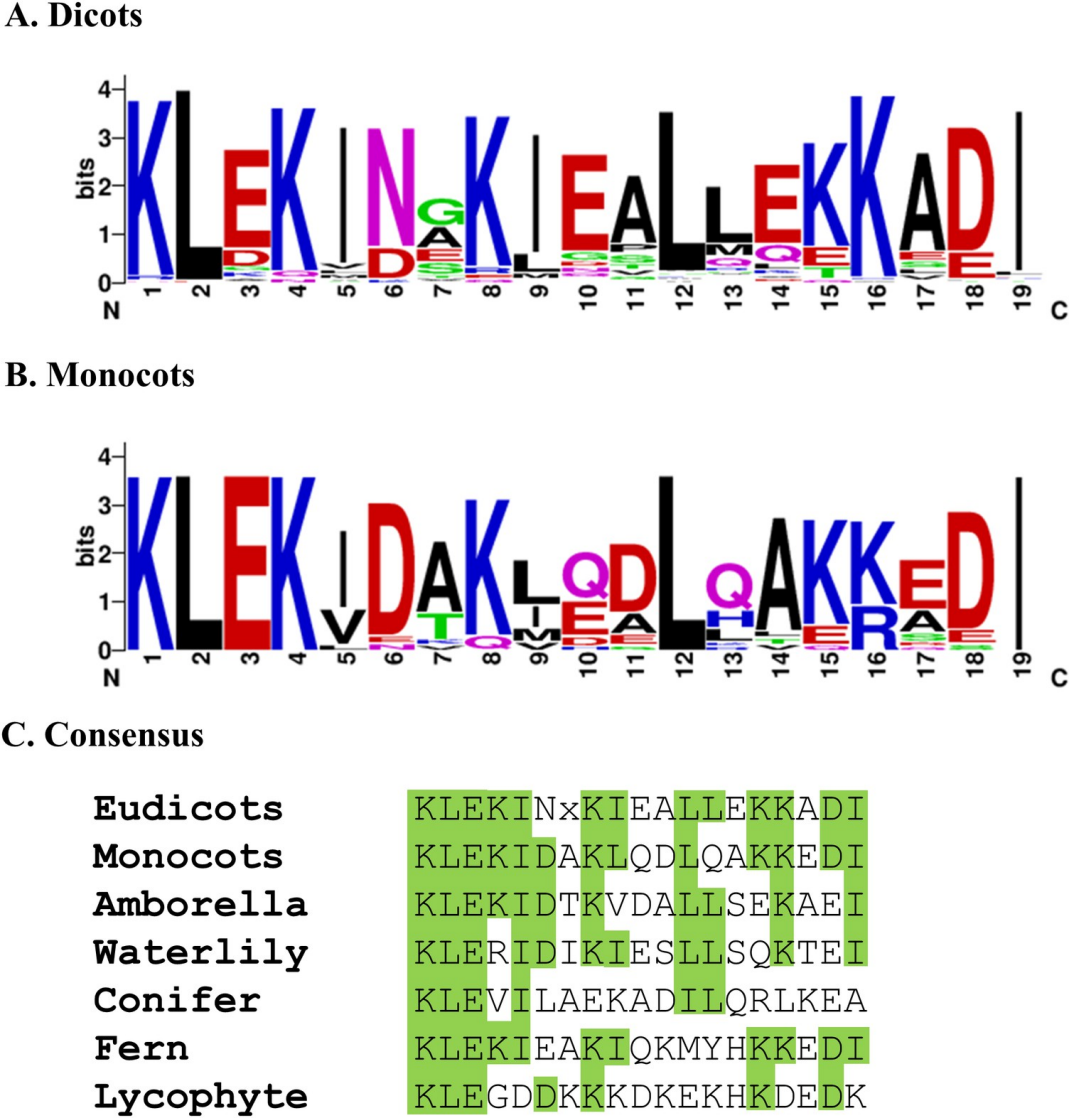
Conserved 19-amino acid domain located near C-terminus of KED proteins in vascular plants (Tracheophyta). Pictograms of the conserved domains are constructed from 68 eudicot species (**A**) and 19 monocot species (**B**). **C**. The consensus sequences for dicots and monocots are compared to those of basal angiosperms *Amborella trichopoda* and waterlily *Nymphaea* spp., a conifer (gymnosperm, *Picea glauca*), a fern (*Adiantum capillus-veneris*) and a lycophyte (*Selaginella moellendorffii*). Most conserved residues are shaded in green. L and I are considered synonymous residues in this study.

Besides the common feature of abundant K, E and D residues, a conserved 19-residue domain near the C-terminus was identified among all 93 vascular plant species examined (Fig 2; a complete list of domain sequences is shown in S3 Table in S1 File). This domain sequence identity is correlated to the taxonomical relation of the species. Thus, all eudicots share a high degree of domain sequence identity (Fig 2A), so do the monocots (Fig 2B). Within eudicots, 10 genera of the Fabaceae family share 58% to 79% sequence identity of this domain, while in monocots 10 genera of the Poaceae family share 58% to 90% sequence identity (S3 Table in S1 File). Within this domain, the first 5 amino acids (consensus KLEKI) and, to a lesser extent, the last 5 amino acids are particularly conserved among all the available KED gene sequences across angiosperm, gymnosperm, fern and lycophyte (Fig 2C). Although the role of this 19-amino acid domain is unknown, the fact of its universal presence in the C-terminal region of all identified KED proteins of vascular plants provides a strong support to the notion that these KED genes are homologous and share the evolutionary lineage.

### Divergent *KED* genes are found in genomes of Bryophytes

**Fig 3:**
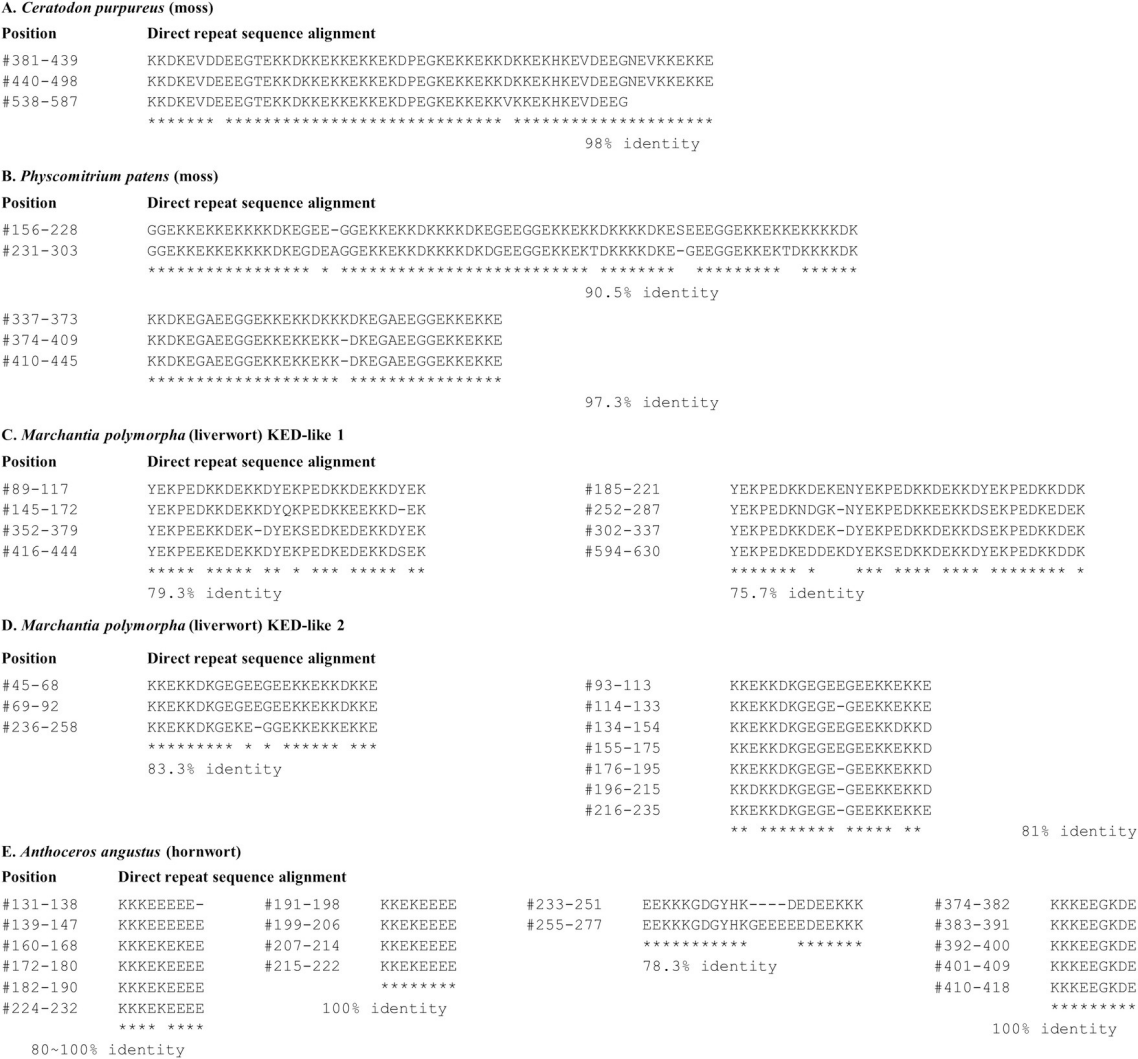
Distribution and identity of direct repeats in bryophyte KED-rich proteins. **A** and **B**, two moss species; **C** and **D**, two isoforms from a liverwort species; **E**, a hornwort species. Locations of the repeats within each protein are indicated by residue positions in the sequence. “-” is gap introduced to maximize the alignment. * denotes identical residues.

We also found protein sequences highly rich in K, E and D in two moss species (*Physcomitrium patens* and *Ceratodon purpureus*), one liverwort (*Marchantia polymorpha*) and one hornwort (*Anthoceros angustus*) (Table 1). Their [K+E+D] residue contents range in 67.9% to 79.1% (Table 1, S2 Table in S1 File). However, except for their high [K+E+D] residue contents, these sequences do not share noticeable sequence similarity with the vascular plant KEDs described above, nor among themselves. Yet, one striking common feature shared by the moss, liverwort and hornwort is that they all possess highly identical direct repeats, mostly made of K and E (Fig 3). For example, the moss *C*. *purpureus* KED has two 59-amino acid regions and one 50-amino acid region with 98% sequence identity to each other, therefore forming a nearly perfect direct repeat (Fig 3A). Likewise, multiple high identity direct repeats are also present in the moss *P*. *patens* KED (Fig 3B). More strikingly, the two separate KED sequences from the liverwort *M*. *polymorpha* are almost entirely made of direct repeats (Fig 3C and 3D), with the exception of the N-terminus. In one *M*. *polymorpha* KED, the sequence YEKPE(D/E)KKDEK repeated 9 times (Fig 3C). Similarly, multiple high identity direct repeats are found in the hornwort *A*. *angustus* KED-rich protein (Fig 3E). By contrast, vascular plant KEDs do not possess such long-length and high-identity repeats. These observations suggest that the bryophyte KED-rich genes examined here underwent divergent or convergent evolution processes and probably do not share the vascular plant (Tracheophyta) *KED* gene lineage.

### KED-rich or KE-rich genes are detected in Charophyta but not in Chlorophyta

**Fig 4:**
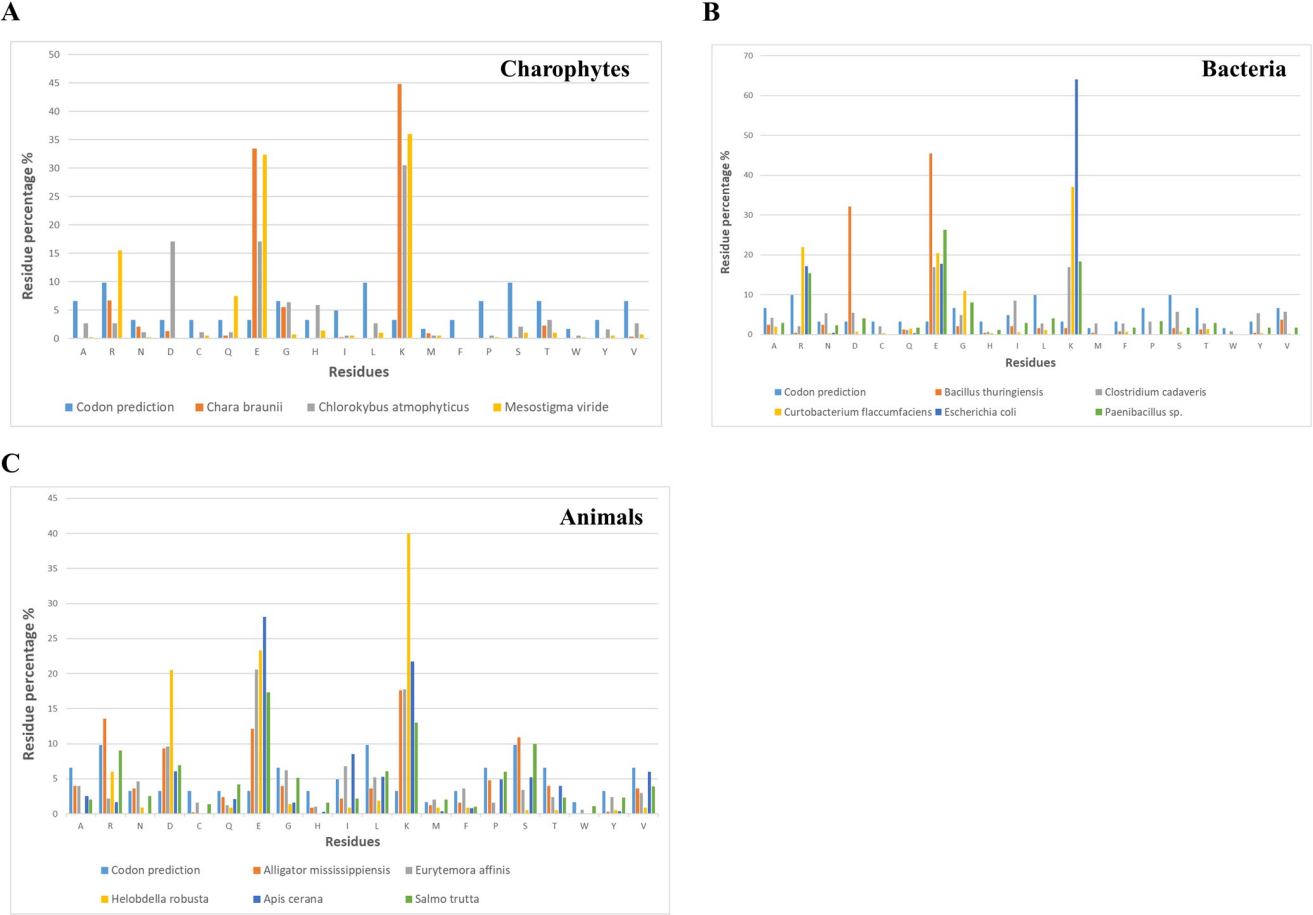
Residue percentage in several representative KED-rich proteins from evolutionarily diverse genomes. Percentage of each residue (in light blue) hypothetically predicted from available codons out of total 61 sense codons is compared to the percentage of each residue in the protein sequences. **A**, charophyte species; **B**, bacterial species; **C**, animal species.

A KED-rich sequence was detected in charophyte *Chlorokybus atmophyticus* (30.5% K, 17.1% E and 17.1% E). Sequences rich in K and E, but not D, were found in charophytes *Chara braunii* (78.2% K+E; 1.3% D) and *Mesostigma viride* (68.4% K+E; 0% D) (Fig 4A). These charophyte sequences show no similarity to the KED sequences of either vascular plants or bryophytes, other than high residue percentages of K and E. We could not find KED-rich sequences in the chlorophyte *Chlamydomonas reinhardtii*.

These observations may suggest that along the lineage of the green plants Viridiplantae, DNA sequences coding for KED- or KE-rich proteins evolved divergently between Charophyta and Embryophyta. Within Embryophyta, these genes continued to diverge between Tracheophyta and Bryophytes, leading to a wide spread of the proteins that are highly enriched with K, E and D residues but are also distinct between these two clades.

### Sequences rich in KED residues are found in some bacterial and animal genomes

DNA sequences coding for proteins with unusually high contents of K, E or D residues were also detected in some bacterial species, although none of them is rich in all these three residues (Fig 4B). For example, the *Bacillus thuringiensis* protein is ED-rich (1.6% K, 45.5% E, 32.1% D), whereas the sequences from *Curtobacterium flaccumfaciens* (37% K, 20.4% E, 0.6% D) and *Escherichia coli* (64.1% K, 17.7% E, 0% D) are both KE codon-rich. There is no noticeable similarity in sequence alignment between these bacterial sequences.

By contrast, sequences highly enriched with all K, E and D residues were detected in the genomes of five diverse animal species of Nephrozoa, belonging to Vertebrata-Euteleostomi (*Alligator mississippiensis* and *Salmo trutta*), Ecdysoza (*Eurytemora affinis* and *Apis cerana*) and Lophotrochozoa (*Helobdella robusta*, with an astonishing 83.7% [K+E+D]) (Fig 4C, S4 Table in S1 File). There is no considerable sequence similarity between these animal KED proteins, besides their high abundance of K, E and D residues. KED-rich proteins are likely present in other animal species that were not included in this study.

## Discussion

In the public databases, many of those retrieved KED sequences are annotated with names of suggestive function without experimental evidence. For example, these KEDs are variously called DNA ligase 1, cylicin-2, myb-like protein X, ABC transporter F family member 4-like and several others. On the other hand, our analysis using I-TASSER computer simulation [17] of the tomato KED amino acid sequence predicted a linear, un-structured conformation (S3 Fig in S1 File), implicating none of enzymatic or signal transducing activity for this protein. Although some sequence segments in the KED protein might be perceived certain function by computational algorithms, it is likely that most of these designations are misnomers or misleading. Both the previous report [6] and our studies (Fig 1) have shown that *KED* gene is wound inducible. We have shown that this pattern of gene expression is conserved at least across seed plants (angiosperms and gymnosperms), as demonstrated by the RT-qPCR for several representative plants (Fig 1). However, currently the biological function of KED proteins is still not known, and its proper and more informative name remains to be coined.

There is no indication that any of these bacterial or animal KED-rich sequences (Fig 4B and 4C) share evolutionary history with plant KEDs, which could be a case of convergent evolution. Nevertheless, the seemingly random/erratic occurrences of proteins with unusually high [K+E+D] content encoded by unrelated bacterial and animal genomes (Fig 4) highlight the structural and functional importance of the amino acid residues K, E and D experiencing codon enrichment during divergent gene evolution, likely a similar scenario for plant KEDs as well.

**Fig 5:**
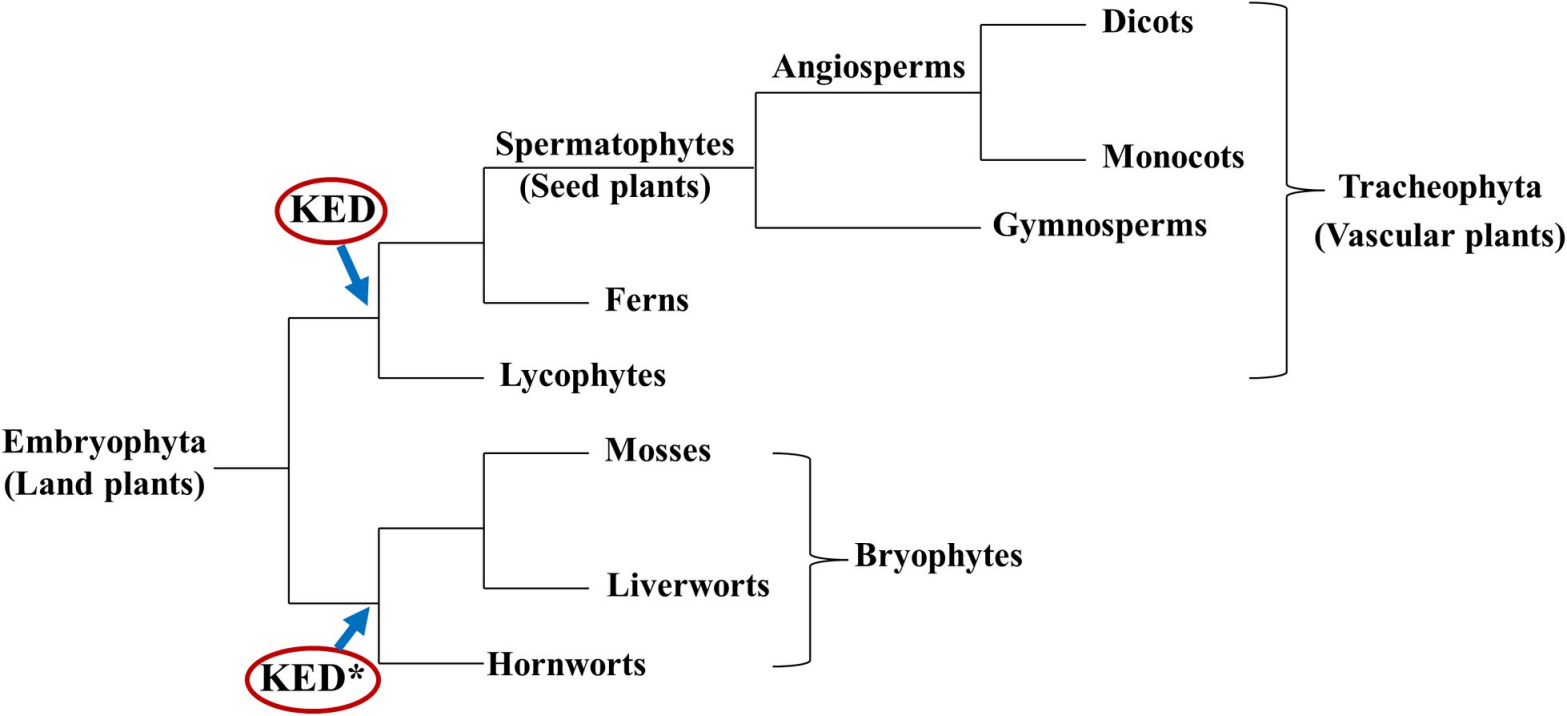
Proposed emergence of *KED* genes along taxonomic lineages of land plants. The *KED* genes likely appeared after emergence of vascular plants ca 450 mya and are conserved throughout Tracheophyta (**KED**), which are distinct from the KED-rich protein genes (KED*) found in mosses, liverworts and hornworts. The classification diagram is adapted from [21]. Branch length is not scaled to divergent distance.

It is generally accepted that plants started to colonize land some 450 ~ 600 million years ago (mya) [3, 18] when a single lineage of the freshwater charophyte green algae set “foot” on land [18, 19]. Thereafter, the land plants (Embryophyta) have evolved into diverse lineages (Fig 5) including the emergence of vascular plants (Tracheophyta) about 450 mya and seed plants (spermatophytes) some 320 mya [1–3, 20]. Our searching of available genome sequences did not detect a KED-rich sequence in charophyte species that matches the ones from the land plants (Embryophyta), suggesting that the ancestral *KED* genes likely appeared after land plants emerged. Furthermore, from available genomic data, we could detect homologous *KED* genes only in vascular plants (Tracheophyta) including angiosperms, gymnosperms, a fern and a lycophyte, all of which share a conserved signature domain (Fig 2). Yet the KED-rich sequences found in other Embryophyta group of Bryophytes show no significant similarity to the vascular plant KEDs. Here, two simplistic explanations can be speculated. One is that a common ancestor KED-rich gene for all land plants (Embryophyta) underwent divergent evolution pathways, forming two different KED-rich gene lineages between Tracheophyta and Bryophytes. Another possibility is that two independent evolution (convergent) processes prompted the emergence of two separate *KED* gene ancestors: one for Tracheophyta and one for Bryophytes, resulting in distinct extant tracheophyte KED group and bryophyte KED group (Fig 5). To further understand the *KED* gene evolutionary process, more genomic and functional investigations are needed. For example, whether the bryophytes share the similar *KED* gene expression patterns with the tracheophyte plants, such as response to mechanical (wounding) stresses (Fig 1), should provide some clues of their functional conservation.

On the other hand, KED-rich or KE-rich sequences were also detected in charophyte species (Fig 4A), but not in chlorophyte. This may suggest that the genomic evolutionary process of codon enrichment for K, E and D started probably long before the emergence of land plants (Embryophyta), but not at the appearance of green plants (Viridiplantae). The divergence of Embryophyta from Charophyta led to distinct gene lineages of KED-rich coding sequences. This K-E-D codon enrichment process may have also independently taken place during evolution of bacteria and animals, resulting in DNA sequences coding for some proteins with extraordinarily high abundance of K, E and D (Fig 4B and 4C).

We propose (Fig 5) that the ancestor *KED* genes emerged after establishment of land plants (Embryophyta) ca 450 mya. Thereafter, the *KED* genes diverged between vascular plants and bryophytes to form distinct KED classes of present-day vascular plant KED and bryophyte KED* as illustrated in Fig 5. While the vascular plant KEDs share a conserved 19-residue domain (Fig 2) and the bryophyte KED sequences (Fig 3) are populated with direct repeats, these two classes share little sequence similarity to each other, besides high contents of K, E and D.

Each of the 20 canonical amino acids constituting proteins has one to six designated codons from the gene’s coding sequence. Overall, the number of synonymous codons for an amino acid somewhat correlates with its abundance in the protein composition. For example, leucine (L), which is encoded by 6 different codons, is the most abundant amino acid in protein databases, whereas the single codon-encoded tryptophan (W) is the least abundant [22]. K, E and D each is encoded by 2 synonymous codons. Thus in theory, the expected percentage of K, E or D in a protein would be 3.28% each (Fig 4). However, in reality, relative amino acid abundance can be influenced by codon bias [13], metabolic cost of amino acid biosynthesis and amino acid decay rates [14, 23–25]. Analysis of proteomes of over 120 species showed the average relative abundance of up to 7.14% (K), 7.34% (E) and 5.62% (D) [14, 26, 27], which is approximately twice the abundance estimated by the genetic code. Then, why have the KED proteins acquired so many K, E and D residues (as much as 79% [K+E+D] in plants), at the expense of other 17 amino acids, when in an average protein [K+E+D] would account around 20%? Since the structure and function of a protein is primarily determined by the interactions among its amino acid residues, it is reasonable to assume that the extraordinarily high enrichment of K, E and D in these proteins must have biological implications.

Due to its chemical structure, lysine residues are known to often play some unique roles in protein structure and stability [28, 29]. For example, K has a very high nucleophilicity, the ability of its side chain to form a covalent band with other molecules [14, 30]. Thus, K has been shown to be important in the crosslinking between three helical polypeptides in collagen, resulting in its stability and tensile strength [31, 32]. Likewise, K residues are critical to the formation of peptidoglycan crosslinks, thus the architecture and stability of the bacterial cell walls [33]. All plant *KED* genes examined so far are wound-induced (Fig 1). In fact, gently brushing the leaves without cell damage was sufficient to trigger *KED* gene transcription (unpublished data). Plant KEDs have a composition of over 25% (up to 37%) K. It can be speculated that these densely-positioned K residues may facilitate a possible crosslinked matrix-like polymer structure, which would contribute to KED’s role in plant defense response to constantly occurring mechanical stress, such as wound healing or membrane strengthening. On the other hand, K, E and D all belong to the group of so-called “disorder-promoting residues” in the intrinsically disordered proteins [34]. Most D/E-rich proteins are predicted to be unstructured and intrinsically disordered [35]. Indeed, our computer simulation analysis of tomato KED confirmed a linear, un-structured conformation (S3 Fig in S1 File). This linear disordered structure with a possibility of lysine-facilitated crosslinks between KED molecules seems feasible to serve a role of a wound “sealer” as a rapid first responder to cell mechanical damage. The conserved pattern of *KED* gene expression in response to wounding among a wide range of vascular plants (Fig 1) may reflect the evolutionary history of plants experiencing unavoidable cellular damages caused by mechanical stress. However, these postulations need further experimentally tested.

Our assessment of plant KED evolution will be enhanced by further discovery and investigation of KED-like sequences in more plant genomes, particularly in Bryophyta, Charophyta and possibly Chlorophyta. Considering the fact that some bacterial and animal species also possess KED-, KE- or ED-rich genes, it should not be surprising if DNA sequences with K-E-D codon enrichment prove to be more prevalent than our current study has revealed. Further genomic sequence analysis and genetic experiments will shed light into the selection force for this group of proteins with a composition extremely biased for K, E and D and its biological or structural significance. More broadly, functional study of these distinct groups of conserved *KED* genes should generate knowledge that strengthens our understanding of gene variation process in association with evolution history of green plants in response to changing environments.

## Conclusions

Genes coding for proteins highly rich in K, E and D residues are present in species from all groups of vascular plants (Tracheophyta) including angiosperm, gymnosperm, fern and lycophyte. These KED proteins share a conserved 19-amino acid domain near the C-terminus, and they are different from other KED-rich, direct repeat-dense proteins from bryophyte species. KED- or KE-rich genes are detected in Charophyta but not in Chlorophyta, whereas unrelated DNA sequences highly enriched with codons for KED, KE or ED are also found in some bacterial and animal species. Our studies highlight the complex evolution pathways for the codon enrichment in the genomes. Evolutionary conservation of the vascular plant KEDs implicates their shared function in response to mechanical and wounding stress.

## Supporting information

S1 File This file contains all the supporting tables and figures. (DOCX)
