# Supplementary material for: Evolutionary analysis of KED-rich proteins in plants
Source: PLoS One. 2023 Mar 8;18(3):e0279772. doi: 10.1371/journal.pone.0279772 (PMC9994729; doi:10.1371/journal.pone.0279772)
Supplement: S1 File — (DOCX) [file pone.0279772.s001.docx]

**Supporting information**

**Evolutionary analysis of KED-rich proteins in plants**

**Table S1.** Sequences of primers used in this study

**Table S2.** Calculated parameters of land plant (Embryophyte) KED-rich proteins

**Table S3**. Conserved domain in vascular plant (Tracheophyte) KED-rich proteins

**Table S4.** Calculated parameters of animal KED-rich proteins

**Figure S1.** MEGA-constructed phylogenetic tree based on amino acid sequences of plant KEDs.

**Figure S2.** MEGA-derived phylogenetic tree based on nucleotide sequences of plant KED coding regions.

**Figure S3**. I-TASSER computer simulation analysis of tomato KED amino acid sequence.

Table S1. Sequences of primers used in this study

| Name | Sequence (5’→ 3’) | Gene |
| --- | --- | --- |
| PGK-1  PGK-2 | TCGTCCGAGTTGATCTCAACGT  AGGCAAGGATGACTCTGGCA | tomato phosphoglycerate kinase, as a reference gene |
| SlK-2  SlK-3 | TCAGCGACTACTAGTCGTGTGCC  TCCAAGTTGGATAAGATCAATGCCAAACT | tomato KED-rich protein |
| AtPP-1  AtPP-2 | CTTCCACCTTTGGAGACTCTATC  CCTCATCTGAGCACCAATTCTA | *Arabidopsis thaliana* protein phosphatase 2A regulatory subunit A, as a reference gene |
| AtPP-3  AtPP-4 | GCACACATTCTTCCTGTGATTG  GGTCCTACAGCTTCACAAAGT | *Arabidopsis thaliana* protein phosphatase 2A regulatory subunit A, as a reference gene |
| AtTIP-1  AtTIP-2 | GATTTGGCTGCTCTCTCACTTA  ACTCCATTGTCAGCCAGTTC | *Arabidopsis thaliana* TIP41-like protein, as a reference gene |
| AtTIP-3  AtTIP-4 | GGTTCCTCCTCTTGCGATTT  ACAGTTGGTGCCTCATCTTC | *Arabidopsis thaliana* TIP41-like protein, as a reference gene |
| AtKED-7  AtKED-8 | CCATGGAGATCTGGAGGTTAAAG  CTTCTTCTAACTCCTCGTGTTTCT | *Arabidopsis thaliana* KED-rich protein |
| AtKED-9  AtKED-10 | GGGAAAGAAAGGAAAGGGAGAG  TTATGATCGGCGGCTTCATC | *Arabidopsis thaliana* KED-rich protein |
| AtKED-11  AtKED12 | GGGTGGTAATGACATCGGTAAG  CTCTCCCTCTGCGTCTTTAATC | *Arabidopsis thaliana* KED-rich protein |
| ZmEF-1  ZmEF-2 | AGGCCCGTTATGATGAGATTG  ACCAGAGATTGGAACGAAGTG | *Zea mays* elongation factor 1-alpha, as a reference gene |
| ZmEF-3  ZmEF-4 | GTATCCTCCTCTTGGTCGATTTG  GTCCTTCTTCTCCACACTCTTG | *Zea mays* elongation factor 1-alpha, as a reference gene |
| ZmTub-1  ZmTub-2 | CAAGTTCTGGGAGGTGATCTG  CTCGTTGTAGTAGACGTTGATCC | *Zea mays* beta tubulin 4, as a reference gene |
| ZmTub-3  ZmTub-4 | CAACGAGGCGCTCTATGATATT  GAGATCAGATGGTTCAGGTCAC | *Zea mays* beta tubulin 4, as a reference gene |
| ZmKED-1  ZmKED-2 | GAAGTCGGTGGAGCATGAATA  CCTTCCCTGAATCTCCATCATC | *Zea mays* KED-rich protein (XM_008653830) |
| ZmKED-3  ZmKED-4 | CAAGGGAAACGCGAATGAAG  CTCTCCAACCTGTTCCAGTATC | *Zea mays* KED-rich protein (XM_008653830) |
| ZmKED-5  ZmKED-6 | AAGGACAAGGGTGAGAAGAATG  CTCTCTTGGCATGTAGGTCTTG | *Zea mays* KED-rich protein (NM_001320969) |
| ZmKED-7  ZmKED-8 | AGGAGGTCGAGGTAGAGATTG  CCTTCTTCTTGGTACCCTTTCC | *Zea mays* KED-rich protein (NM_001320969) |
| PgEF-1  PgEF-2 | AAGGACGGTCAGACTAGAGAA  GCCTTTGAGTACTTGGGAGTAG | *Picea glauca* elongation factor-1 alpha, as a reference gene |
| PgEF-3  PgEF-4 | CTGGAGAAGGAACCCAAGTTT  GAGGATACTCAGCGAAGGTTTC | *Picea glauca* elongation factor-1 alpha, as a reference gene |
| PgK-1  PgK-2 | GATGCCAAGCTGGAGGTAAT  GCTCCACCGTATGATCAATCT | *Picea glauca* KED-rich protein |
| PgK-3  PgK-4 | AGTCAGAGTCCCTTGGAGAT  CAACATCAGTTGCCTCTGTTTC | *Picea glauca* KED-rich protein |
| PgK-5  PgK-6 | ACTGATGTTGGACAGCATGTA  CTCCTTATCCTTGTCCTTCTCTTT | *Picea glauca* KED-rich protein |
| NmpA1  NmpA2 | ATGCTCCAAGGGCAGTATTC  CTGGGCTTCATCACCAACATA | *Nymphaea colorata* actin, as a reference gene |
| NmpA3  NmpA4 | CTGCCATTGAGAAGAGCTATGA  AATGAGAGATGGCTGGAACAG | *Nymphaea colorata* actin, as a reference gene |
| NmpK1  NmpK2 | CAAGGGAAGTGCAGGAGAAT  CTCGGTTCGTCTTTCTCATCTC | *Nymphaea colorata* KED-rich protein |
| NmpK3  NmpK4 | GTGAACATGAAGGAGACGAGAA  TCCTTTGCCTTTGCCTCTT | *Nymphaea colorata* KED-rich protein |
| NmpK5  NmpK6 | GAGCTGCACATCAAAGGTAAGA  CCATCCTTCCGCACAGATTT | *Nymphaea colorata* KED-rich protein |

Table S2. Calculated parameters of land plant (Embryophyte) KED-rich proteins

| **Species (Common Name)** | **GenBank Accession #** | **Molecular Weight (Dalton)** | **Amino Acid Number** | **K%** | **E%** | **D%** | **KED%** | **PI** |
| --- | --- | --- | --- | --- | --- | --- | --- | --- |
| **Angiosperm: Eudicots** | | | | | | | | |
| *Vigna angularis* (Adzuki bean) | XM_017560437 | 58102 | 498 | 30.9 | 18.9 | 13.7 | 63.5 | 7.09 |
| *Vigna radiata* (Mung bean) | XM_014635587 | 61993 | 530 | 30.2 | 19.1 | 12.6 | 61.9 | 8.29 |
| *Vigna unguiculata* (Cowpea) | XM_028074767 | 52114 | 445 | 30.8 | 20.2 | 11.7 | 62.7 | 7.86 |
| *Arachis duranensis* (Herb) | XM_021134265 | 37069 | 317 | 33.1 | 21.1 | 9.5 | 63.7 | 9.02 |
| *Arachis hypogaea* (Peanut) | XM_025768881 | 41218 | 351 | 34.2 | 21.4 | 10.3 | 65.9 | 9.00 |
| *Arachis ipaensis* (Wild peanut) | XM_016325710 | 40861 | 349 | 34.1 | 21.5 | 9.7 | 65.3 | 9.00 |
| *Cicer arietinum* (Chickpea) | XM_027331867 | 49846 | 428 | 33.9 | 19.9 | 13.1 | 66.9 | 8.77 |
| *Prosopis alba* (White carob tree) | XM_028941941 | 45996 | 395 | 33.9 | 21.8 | 13.7 | 69.4 | 7.47 |
| *Glycine max* (Soybean) | XM_006598911 | 54343 | 469 | 29.4 | 17.3 | 12.4 | 59.1 | 8.46 |
| *Glycine soja* (Wild soybean) | XM_028362450 | 45537 | 388 | 35.8 | 22.2 | 13.4 | 71.4 | 8.35 |
| *Phaseolus vulgaris* (Common bean) | XM_007161897 | 47107 | 407 | 29.0 | 17.0 | 12.5 | 58.5 | 8.65 |
| *Lupinus angustifolius* (Lupin) | XM_019597524 | 78250 | 673 | 37.0 | 22.4 | 14.1 | 73.5 | 8.32 |
| *Cajanus cajan* (Pigeon pea) | XM_020356508 | 52159 | 446 | 31.6 | 18.4 | 13.5 | 63.5 | 8.73 |
| *Medicago truncatula* (Barrel medic) | BT144475 | 27835 | 238 | 35.3 | 20.2 | 15.1 | 70.6 | 8.12 |
| *Abrus precatorius* (Rosary pea) | XM_027503355 | 46454 | 394 | 32.0 | 20.8 | 13.2 | 66.0 | 7.51 |
| *Prunus dulcis* (Almond) | AP019303 | 41334 | 350 | 32.9 | 19.4 | 16.3 | 68.6 | 6.76 |
| *Prunus persica* (Peach) | XM_020569208 | 57471 | 486 | 32.7 | 21.0 | 14.0 | 67.7 | 6.71 |
| *Prunus mume* (Plum blossom) | XM_008242281 | 55554 | 473 | 32.8 | 20.7 | 13.7 | 67.2 | 6.91 |
| *Prunus avium* (Sweet cherry) | XM_021977203 | 57164 | 487 | 32.8 | 20.2 | 14.2 | 67.2 | 7.58 |
| *Malus domestica* (Apple) | XM_029092494 | 61427 | 526 | 33.7 | 20.7 | 14.4 | 68.8 | 6.87 |
| *Pyrus x bretschneideri* (Pear) | XM_018646758 | 60920 | 524 | 33.4 | 20.2 | 13.7 | 67.3 | 7.87 |
| *Fragaria vesca* (Wild strawberry) | XM_004288360 | 46379 | 397 | 29.7 | 20.7 | 10.6 | 61.0 | 7.91 |
| *Rosa chinensis* (China rose) | XM_024331981 | 44623 | 380 | 29.2 | 20.3 | 10.0 | 59.5 | 8.81 |
| *Morus notabilis* (Mulberry) | XM_024168993 | 65034 | 551 | 29.9 | 22.0 | 10.0 | 61.9 | 8.06 |
| *Cannabis sativa* (Marijuana) | XM_030648096 | 51922 | 443 | 35.0 | 22.3 | 13.1 | 70.4 | 7.50 |
| *Ziziphus jujuba* (Red date) | XM_025066998 | 53437 | 446 | 36.3 | 22.9 | 13.2 | 72.4 | 8.56 |
| *Ricinus communis* (Castor bean) | XM_015723337 | 45421 | 385 | 33.5 | 22.9 | 11.7 | 68.1 | 7.98 |
| *Hevea brasiliensis* (Rubber tree) | XM_021830341 | 47708 | 402 | 34.3 | 26.1 | 9.7 | 70.1 | 7.92 |
| *Manihot esculenta* (Yuca/cassava) | XM_021763985 | 49589 | 423 | 32.9 | 23.2 | 11.1 | 67.2 | 7.54 |
| *Populus trichocarpa* (Black cottonwood) | XM_024583662 | 52276 | 450 | 32.2 | 23.3 | 9.8 | 65.3 | 7.86 |
| *Populus euphratica* (Desert poplar) | XM_011036101 | 60864 | 519 | 33.7 | 23.9 | 9.1 | 66.7 | 8.68 |
| *Quercus suber* (Cork oak) | XM_024070628 | 56956 | 484 | 31.6 | 22.7 | 11.2 | 65.5 | 6.69 |
| *Quercus lobata* (Valley oak) | XM_031111392 | 56914 | 484 | 31.4 | 22.7 | 11.0 | 65.1 | 6.68 |
| *Cucumis sativus* (Cucumber) | XM_031882681 | 69558 | 594 | 34.7 | 22.2 | 10.3 | 67.2 | 9.04 |
| *Momordica charantia* (Bitter melon) | XM_022299800 | 46323 | 396 | 32.8 | 22.0 | 12.1 | 66.9 | 7.50 |
| *Rhodamnia argentea* (White myrtle) | XM_030659579 | 62051 | 530 | 27.4 | 20.8 | 10.6 | 58.8 | 7.24 |
| *Syzygium oleosum* (Blue cherry) | XM_030597036 | 61232 | 526 | 27.4 | 20.2 | 10.8 | 58.4 | 7.57 |
| *Eucalyptus grandis* (Flooded Gum tree) | XM_018864849 | 58892 | 513 | 22.6 | 22.2 | 6.6 | 51.4 | 5.55 |
| *Punica granatum* (Pomegranate) | XM_031526535 | 50394 | 437 | 30.2 | 24.5 | 9.2 | 63.9 | 5.87 |
| *Theobroma cacao* (Cacao tree) | XM_018121055 | 89937 | 740 | 36.8 | 28.5 | 9.6 | 74.9 | 8.26 |
| *Durio zibethinus* (Durian) | XM_022892162 | 50511 | 426 | 31.7 | 27.9 | 8.2 | 67.8 | 5.68 |
| *Gossypium raimondii* (Cotton) | XM_012621289 | 63782 | 532 | 34.8 | 24.6 | 10.9 | 70.3 | 7.48 |
| *Gossypium hirsutum* (Mexican cotton) | XM_016869005 | 65884 | 551 | 34.3 | 24.1 | 11.8 | 70.2 | 6.49 |
| *Gossypium arboreum* (Tree cotton) | XM_017768549 | 65781 | 550 | 34.5 | 24.0 | 11.8 | 70.3 | 6.85 |
| *Carica papaya* (Papaya) | XM_022042554 | 35103 | 296 | 33.1 | 25.0 | 9.5 | 67.6 | 8.63 |
| *Arabidopsis thaliana* (Thale cress) | NM_001333792 | 60749 | 522 | 33.3 | 22.6 | 10.2 | 66.1 | 8.22 |
| *Arabidopsis lyrata* (Sand cress) | XM_002891944 | 62597 | 537 | 33.3 | 22.7 | 9.7 | 65.7 | 8.53 |
| *Brassica napus* (Rape) | XM_013873451 | 61115 | 524 | 33.6 | 21.9 | 11.1 | 66.6 | 8.53 |
| *Brassica oleracea* (Broccoli) | XM_013757383 | 62273 | 533 | 32.3 | 22.5 | 11.3 | 66.1 | 6.67 |
| *Brassica rapa* (Field mustard) | XM_009115224 | 59222 | 507 | 33.5 | 21.5 | 11.4 | 66.4 | 8.55 |
| *Pistacia vera* (Pistachio) | XM_031398623 | 44012 | 374 | 33.2 | 24.6 | 8.6 | 66.4 | 8.60 |
| *Citrus sinensis* (Sweet orange) | XM_006479072 | 40603 | 345 | 32.2 | 21.4 | 11.3 | 64.9 | 8.54 |
| *Vitis vinifera* (Grape vine) | XM_002266326 | 44898 | 382 | 32.5 | 26.7 | 6.0 | 65.2 | 8.50 |
| *Coffea arabica* (Arabian coffee) | XM_027211368 | 55972 | 480 | 33.1 | 20.8 | 12.1 | 66.0 | 8.37 |
| *Coffea eugenioides* (Coffee bean) | XM_027321431 | 55839 | 479 | 32.8 | 20.5 | 12.7 | 66.0 | 8.23 |
| *Capsicum annuum* (Pepper) | XM_016699553 | 66637 | 572 | 32.7 | 23.6 | 11.7 | 68.0 | 5.69 |
| *Nicotiana tabacum* (Tobacco) | NM_001326188 | 61019 | 513 | 34.7 | 25.0 | 12.5 | 72.2 | 5.83 |
| *Solanum lycopersicum* (Tomato) | XM_004249331 | 50532 | 427 | 34.7 | 24.6 | 11.0 | 70.3 | 6.57 |
| *Solanum pennellii* (Wild tomato) | XM_027912514 | 57709 | 490 | 35.3 | 24.9 | 10.6 | 70.8 | 7.85 |
| *Solanum tuberosum* (Potato) | XM_015310060 | 58784 | 497 | 34.6 | 25.8 | 9.5 | 69.9 | 8.48 |
| *Ipomoea nil* (Morning glory) | XM_019331536 | 50320 | 425 | 32.9 | 22.1 | 12.0 | 67.0 | 8.12 |
| *Olea europaea* (Olive) | XM_023002055 | 50875 | 435 | 31.3 | 21.1 | 10.6 | 63.0 | 8.23 |
| *Erythranthe guttatus* (Seep monkey flower) | XM_012973342 | 32769 | 285 | 30.5 | 23.2 | 10.5 | 64.2 | 5.62 |
| *Sesamum indicum* (Sesame) | XM_011095872 | 70753 | 609 | 31.5 | 21.8 | 9.4 | 62.7 | 8.54 |
| *Daucus carota* (Carrot) | XM_017391791 | 49500 | 423 | 33.1 | 24.3 | 9.7 | 67.1 | 6.49 |
| *Camellia sinensis* (Tea plant) | XM_028247325 | 63792 | 543 | 32.8 | 24.9 | 8.7 | 66.4 | 7.51 |
| *Papaver somniferum* (Opium poppy) | XM_026562616 | 49784 | 427 | 31.9 | 20.4 | 11.5 | 63.8 | 8.17 |
| *Nelumbo nucifera* (Sacred lotus) | NW_010729119 | 27605 | 231 | 35.1 | 23.4 | 10.0 | 68.5 | 8.71 |
|  |  |  |  |  |  |  |  |  |
| **Angiosperm: Monocots** | | | | | | | | |
| *Oryza brachyantha* (African rice) | XM_006657411 | 45931 | 401 | 29.4 | 19.2 | 11.5 | 60.1 | 8.19 |
| *Oryza sativa* (Asian rice) | XM_015791192 | 31515 | 275 | 29.5 | 19.6 | 12.0 | 61.1 | 5.96 |
| *Triticum aestivum* (Wheat) | AK457069 | 46497 | 406 | 31.5 | 17.7 | 13.3 | 62.5 | 8.69 |
| *Aegilops tauschii* (Tausch's goatgrass) | XM_020317031 | 43352 | 379 | 31.7 | 16.6 | 13.7 | 62.0 | 8.86 |
| **Hordeum vulgare* (Barley) | AY137518 |  | 96 | 26.0 | 16.7 | 6.2 | 48.9 |  |
| *Brachypodium distachyon* (Stiff brome) | XM_003557505 | 46232 | 409 | 30.3 | 16.1 | 14.4 | 60.8 | 8.53 |
| *Digitaria exilis* (White fonio) | LR792822 | 45533 | 401 | 29.4 | 16.7 | 13.5 | 59.6 | 7.15 |
| *Setaria italica* (Foxtail millet) | XM_004955483 | 47154 | 418 | 29.9 | 17.7 | 13.4 | 61.0 | 6.49 |
| *Setaria viridis* (Green foxtail) | XM_034722522 | 47154 | 418 | 29.9 | 17.7 | 13.4 | 61.0 | 6.49 |
| *Panicum hallii* (Halls panicgrass) | XM_025946102 | 49239 | 431 | 30.6 | 18.6 | 13.0 | 62.2 | 7.07 |
| *Zea mays-1* (Corn) | NM_001320969 | 31543 | 283 | 27.2 | 17.7 | 11.7 | 56.6 | 6.97 |
| *Zea mays-2* (Corn) | NM_001320969 | 24477 | 224 | 25.4 | 16.5 | 9.8 | 51.7 | 8.28 |
| *Sorghum bicolor* (Great millet) | XM_021452805 | 48674 | 431 | 29.9 | 16.9 | 13.9 | 60.7 | 7.54 |
| *Ananas comosus* (Pineapple) | XM_020240651 | 69265 | 594 | 31.8 | 21.4 | 12.3 | 65.5 | 6.88 |
| *Dendrobium catenatum* (Lithophytic orchid) | XM_020821417 | 64548 | 547 | 31.3 | 21.2 | 11.7 | 64.2 | 7.81 |
| *Phalaenopsis equestris* (Orchid) | XM_020724026 | 46938 | 401 | 33.9 | 20.7 | 13.2 | 67.8 | 8.33 |
| *Phoenix dactylifera* (Date palm) | XM_008779646 | 59828 | 515 | 31.5 | 21.2 | 11.7 | 64.4 | 7.14 |
| *Elaeis guineensis* (Oil palm) | XM_010909609 | 56032 | 482 | 31.3 | 20.1 | 12.2 | 63.6 | 8.25 |
| *Asparagus officinalis* (Asparagus) | XM_020400398 | 46747 | 403 | 32.3 | 25.6 | 9.7 | 67.6 | 6.83 |
| *Musa acuminata* (Banana) | XM_009415598 | 58164 | 500 | 30.4 | 20.6 | 11.2 | 62.2 | 7.50 |
| *Spirodela polyrhiza* (Duckweed) | CP019096 | 36414 | 319 | 20.4 | 23.5 | 5.6 | 49.5 | 8.22 |
|  |  |  |  |  |  |  |  |  |
| **Angiosperm: Basal** | | | | | | | | |
| *Amborella trichopoda* (Amborella) | XM_011627501 | 65880 | 553 | 34.4 | 24.4 | 11.4 | 70.2 | 7.11 |
| *Nymphaea thermarum* (Water lily) | JAANDH010000271 | 56460 | 490 | 26.3 | 20.4 | 6.3 | 53.0 | 8.77 |
| *Nymphaea colorata* (Water lily) | XM_031622050 | 54940 | 476 | 26.5 | 21.4 | 6.3 | 54.2 | 8.65 |
| **Gymnosperm: Conifers** | | | | | | | | |
| *Picea glauca* (White spruce) | BT110330 | 28167 | 240 | 29.2 | 14.2 | 12.9 | 56.3 | 9.05 |
| *Picea sitchensis* (Sitka spruce) | BT122514 | 28150 | 240 | 28.7 | 14.6 | 12.5 | 55.8 | 8.98 |
| ***Cryptomeria japonica* (Japanese cedar) | AK409118 | 21075 | 178 | 34.8 | 16.3 | 20.2 | 71.3 | 6.02 |
| **Fern** | | | | | | | | |
| *Adiantum capillus-veneris* (Venus hair fern) | JABFUD010159261 | 65684 | 569 | 26.7 | 19.3 | 11.1 | 57.1 | 6.16 |
| **Lycophyte** | | | | | | | | |
| *Selaginella moellendorffii* | XM_024678618 | 37229 | 313 | 35.1 | 22.4 | 11.5 | 69.0 | 8.94 |
| **Moss** | | | | | | | | |
| *Physcomitrium patens* | ABEU02000005 | 55098 | 484 | 35.5 | 25.4 | 8.9 | 69.8 | 8.48 |
| *Ceratodon purpureus* | CM026433 | 75984 | 641 | 32.6 | 26.4 | 8.9 | 67.9 | 6.29 |
| **Liverwort** | | | | | | | | |
| *Marchantia polymorpha* | AP019872 | 78952 | 644 | 30.1 | 23.6 | 19.3 | 73.0 | 4.71 |
| **Hornwort** | | | | | | | | |
| *Anthoceros angustus* | VJWM01000089 | 51406 | 425 | 31.8 | 32.5 | 7.1 | 71.4 | 5.24 |

* Partial sequence without N-terminus.

** Partial sequence without C-terminus.

Table S3. Conserved domain in vascular plant (Tracheophyte) KED-rich proteins

| **Taxon Family** | **Species (Common Name)** | **Conserved Domain** |
| --- | --- | --- |
| **Angiosperm: Eudicots** | | |
| Fabaceae | *Vigna angularis* (Adzuki bean) | KLEKINAKIEPLLQKKADI |
|  | *Vigna radiata* (Mung bean) | KLEKINAKIEPLLQKKADI |
|  | *Vigna unguiculata* (Cowpea) | KLEKINAKIEPLLQKKADI |
|  | *Arachis duranensis* (Herb) | KLGKINEKIETLLQKKADI |
|  | *Arachis hypogaea* (Peanut) | KLGKINEKIETLLQKKADI |
|  | *Arachis ipaensis* (Wild peanut) | KLGKINEKIETLLQKKADI |
|  | *Cicer arietinum* (Chickpea) | KLEKMNAKIEALLEKKADI |
|  | *Prosopis alba* (White carob tree) | KLEKINTKLEALRQKKEEI |
|  | *Glycine max* (Soybean) | KLEKINGKIQPLLEKKADI |
|  | *Glycine soja* (Wild soybean) | KLEKINGKIQPLLEKKADI |
|  | *Phaseolus vulgaris* (Common bean) | KLEKINGKLEPLLEKKADI |
|  | *Lupinus angustifolius* (Lupin) | KLEKINGKIEALSEEKATI |
|  | *Cajanus cajan* (Pigeon pea) | KLEKINGKLESLLEKKADI |
|  | *Medicago truncatula* (Barrel medic) | KLEKANAKLEALLEKKADL |
|  | *Abrus precatorius* (Rosary pea) | KLEKINEKIEALLEQKADI |
| Rosaceae | *Prunus dulcis* (Almond) | KLEKINGKIEALRETKVDI |
|  | *Prunus persica* (Peach) | KLEKINGKIEALRETKLDI |
|  | *Prunus mume* (Plum blossom) | KLEKINGKIEALCETKLDI |
|  | *Prunus avium* (Sweet cherry) | KLEKINGKIEALHETKLDI |
|  | *Malus domestica* (Apple) | KLEKINGKIEGLLETKSDI |
|  | *Pyrus x bretschneideri* (Pear) | KLEKINVKIEGLLEKKSDI |
|  | *Fragaria vesca* (Wild strawberry) | KLEKIDGKIEALVAKKADI |
|  | *Rosa chinensis* (China rose) | KLEKINGKIEALVEKKADI |
| Moraceae | *Morus notabilis* (Mulberry) | KLEKINGKIETLLEEKADT |
| Cannabaceae | *Cannabis sativa* (Marijuana) | KLEKINAKIEVLLEQKAEI |
| Rhamnaceae | *Ziziphus jujuba* (Red date) | KLEQIDAKMEALLGKKADI |
| Euphorbiaceae | *Ricinus communis* (Castor bean) | KLDKIDGKINALLEKKADI |
|  | *Hevea brasiliensis* (Rubber tree) | KLQKIDGKIEALLEKKADI |
|  | *Manihot esculenta* (Yuca/cassava) | KLEKIDARIEAMLEKKADI |
| Salicaceae | *Populus trichocarpa* (Black cottonwood) | KLEKINERIEAALEEKDDI |
|  | *Populus euphratica* (Desert poplar) | KLEKINERIEAVLEEKEDI |
| Fagaceae | *Quercus suber* (Cork oak) | KLEQINGKIEALSEKKADI |
|  | *Quercus lobata* (Valley oak) | KLEQINGKIEALLEKKADI |
| Cucurbitaceae | *Cucumis sativus* (Cucumber) | RLEKLDVKINALLLKKVDI |
|  | *Momordica charantia* (Bitter melon) | KLEKIDVKINVLLEKKADI |
| Myrtaceae | *Rhodamnia argentea* (White myrtle) | KLEKINSKIEALLEKKSEI |
|  | *Syzygium oleosum* (Blue cherry) | KLEKLNSQIEALLEKKSEI |
|  | *Eucalyptus grandis* (Flooded Gum tree) | QLEKINGKIEALLEKKAFI |
| Lythraceae | *Punica granatum* (Pomegranate) | KLEKINGQIEALMEKKADI |
| Malvaceae | *Theobroma cacao* (Cacao tree) | RLEKINSKIEALLEKKAEI |
|  | *Durio zibethinus* (Durian) | KLEKINSKIEALLEKKEEI |
|  | *Gossypium raimondii* (Cotton) | KLEKINSKIDALLEKKADI |
|  | *Gossypium hirsutum* (Mexican cotton) | KLEKINTKIDALLEKKADI |
|  | *Gossypium arboreum* (Tree cotton) | KLEKINSKIDALLEKKADI |
| Caricaceae | *Carica papaya* (Papaya) | KEEKIKDKNKEGADKVKDR |
| Brassicaceae | *Arabidopsis thaliana* (Thale cress) | KLAKIDEKIGALMEEKAEI |
|  | *Arabidopsis lyrata* (Sand cress) | KLAKIDEKIGALMEEKAEI |
|  | *Brassica napus* (Rape) | KLKKIDEKIGALMEKKAEI |
|  | *Brassica rapa* (Field mustard) | KLKKIDEKIGALMEKKAEI |
|  | *Brassica oleracea* (Broccoli) | KLKKIDEKIGALMEKKADI |
| Anacardiaceae | *Pistacia vera* (Pistachio) | KLEKINGKIEALMEEKADI |
| Rutaceae | *Citrus sinensis* (Orange) | KLENVNAKIEALIEKKADI |
| Vitaceae | *Vitis vinifera* (Grape vine) | KLDKINAKIEALMEEKADI |
| Rubiaceae | *Coffea arabica* (Arabian coffee) | KLDKVNSEIESLLKKKADI |
|  | *Coffea eugenioides* (Coffee bean) | KLDKVNSEIESLLKKKADI |
| Solanaceae | *Capsicum annuum* (Pepper) | KLDKINAKLEALQQKKADL |
|  | *Nicotiana tabacum* (Tobacco) | KLEKINAKLEALQQKKADI |
|  | *Solanum lycopersicum* (Tomato) | KLDKINAKLEALQLKKADI |
|  | *Solanum pennellii* (Wild tomato) | KLDKINAKLEALQLKKADI |
|  | *Solanum tuberosum* (Potato) | KLDKINAKLEALQLEKADI |
| Convolvulaceae | *Ipomoea nil* (Morning glory) | KLEKINAKMESLEQKRADI |
| Oleaceae | *Olea europaea* (Olive) | KLEKINAKMEALLEKKADI |
| Phrymaceae | *Erythranthe guttatus* (Seep monkey flower) | KLEKINGKIEGLLEKKADI |
| Pedaliaceae | *Sesamum indicum* (Sesame) | KLEKINGKIESLLDKKADI |
| Apiaceae | *Daucus carota* (Carrot) | KLEKINSKIEVLLQEKAEI |
| Theaceae | *Camellia sinensis* (Tea plant) | KLEKYKGKDISKLKTKVEE |
| Papaveraceae | *Papaver somniferum* (Opium poppy) | KLEKMDAKISALQEKKEDI |
| Nelumbonaceae | *Nelumbo nucifera* (Sacred lotus) | KLDNINSKIEVLMEKKADI |
| **Angiosperm: Monocots** | | |
| Poaceae | *Oryza brachyantha* (African rice) | KLEKIDTKIQDLQAKKEDI |
|  | *Oryza sativa* (Asian rice) | KLEKIDTKIQDLQAKKEDI |
|  | *Triticum aestivum* (Wheat) | KLEKIDAKLQDLHAEKEDI |
|  | *Aegilops tauschii* (Tausch's goatgrass) | KLEKIDAKLQDLHAEKEDI |
|  | *Hordeum vulgare* (Barley) | KLEKIDVKIDDLKAKKQEI |
|  | *Brachypodium distachyon* (Stiff brome) | KLEKIDAKLQDLHLEKDDI |
|  | *Digitaria exilis* (White fonio) | KLEKVDTKLQDLQAKREDI |
|  | *Setaria italica* (Foxtail millet) | KLEKVDAKLQDLQAKREDI |
|  | *Setaria viridis* (Green foxtail) | KLEKVDAKLQDLQAKREDI |
|  | *Panicum hallii* (Halls panic grass) | KLEKVDAKLQDLQAKREDI |
|  | *Zea mays-1* (Corn) | KLEKIDAKLHDLQAKREDI |
|  | *Zea mays-2* (Corn) | KLEKVDTKLQDLHAKREDI |
|  | *Sorghum bicolor* (Great millet) | KLEKVDAKLQDLQAKREDI |
| Bromeliaceae | *Ananas comosus* (Pineapple) | KLEKINAKIDALQVKKEEI |
| Orchidaceae | *Dendrobium catenatum* (Lithophytic orchid) | KLEKLDAKMEELQTKKSDI |
|  | *Phalaenopsis equestris* (Orchid) | KLEKIDTKMEELQAKKSDI |
| Arecaceae | *Phoenix dactylifera* (Date palm) | KLEKIDAKVEALLAKKADI |
|  | *Elaeis guineensis* (Oil palm) | KLEKIDAQVEALLAKKADI |
| Asparagaceae | *Asparagus officinalis* (Asparagus) | KLEKIDTQIEALRAKKADI |
| Musaceae | *Musa acuminata* (Banana) | KLEKIDAKMEDLQAKKADI |
| Araceae | *Spirodela polyrhiza* (Duckweed) | KLEKIEKKIESLLAQKASI |
| **Angiosperm: Basal** | | |
| Amborellaceae | *Amborella trichopoda* (Amborella) | KLEKIDTKVDALLSEKAEI |
| Nymphaeaceae | *Nymphaea thermarum* (Water lily) | KLERIDIKIESLLSQKTEI |
| Nymphaeaceae | *Nymphaea colorata* (Water lily) | KLERIDIKIESLLSKKTEI |
| **Gymnosperm: Conifers** | | |
| Pinaceae | *Picea glauca* (White spruce) | KLEVILAEKADILQRLKEA |
|  | *Picea sitchensis* (Sitka spruce) | KMEVILAEKADILQRLKEA |
| **Fern** | | |
| Pteridaceae | *Adiantum capillus-veneris* (Venus hair fern) | KLEKIEAKIQKMYHKKEDI |
| **Lycophyte** |  |  |
| Selaginellaceae | *Selaginella moellendorffii* | KLEGDDKKKDKEKHKDEDK |

Table S4. Calculated parameters of animal KED-rich proteins

| **Species (Common Name)** | **GenBank Accession #** | **Amino Acid #** | **K%** | **E%** | **D%** | **KED%** | **pI** |
| --- | --- | --- | --- | --- | --- | --- | --- |
| *Alligator mississippiensis* (Alligator) | XM_014594096 | 579 | 17.6 | 12.1 | 9.3 | 39.0 | 10.04 |
| *Salmo trutta* (Trout) | XM_029738947 | 830 | 13.0 | 17.3 | 6.9 | 37.2 | 5.68 |
| *Eurytemora affinis* (Copepod) | XM_023478288 | 499 | 17.8 | 20.6 | 9.6 | 48.0 | 4.70 |
| *Apis cerana* (Honeybee) | XM_017057684 | 750* | 21.7 | 28.1 | 6.1 | 56.0 | 4.74 |
| *Helobdella robusta* (Leech) | XM_009012611 | 215 | 40.0 | 23.3 | 20.5 | 83.7 | 8.69 |

* A part (residues 1955 to 2714) of the 6229-amino acid protein microtubule-associated protein futsch.


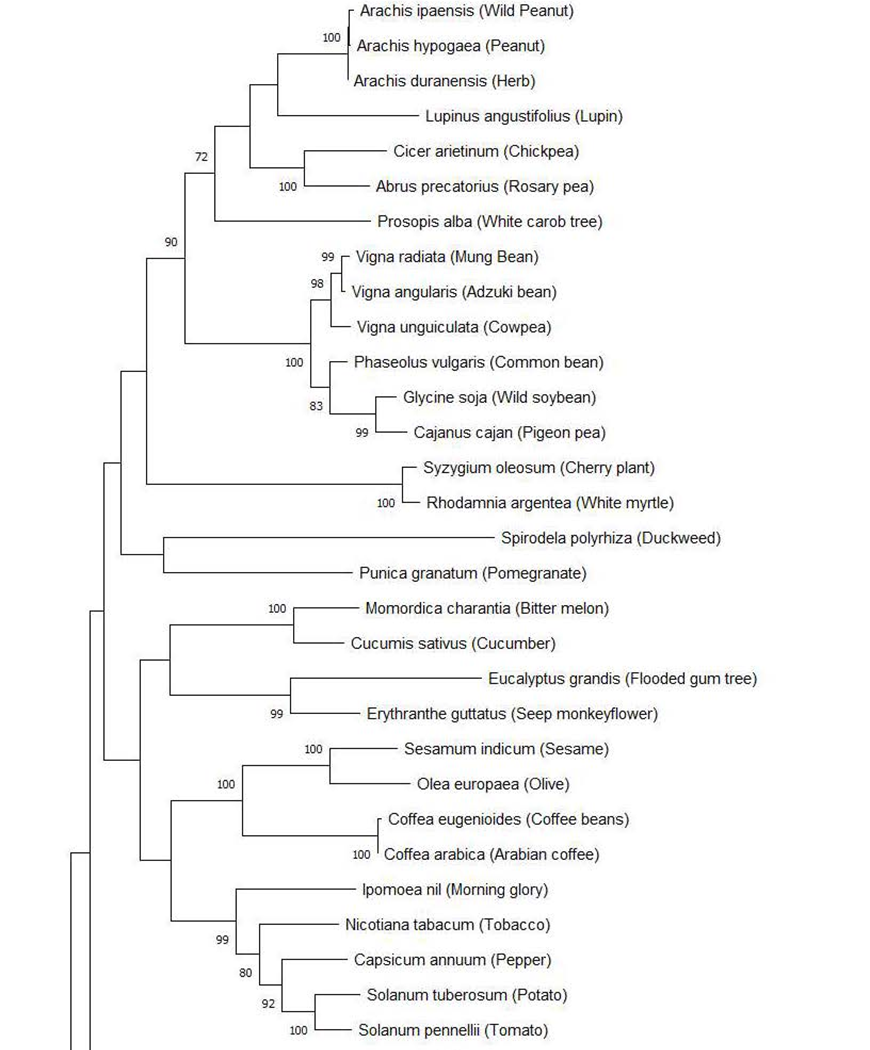


**
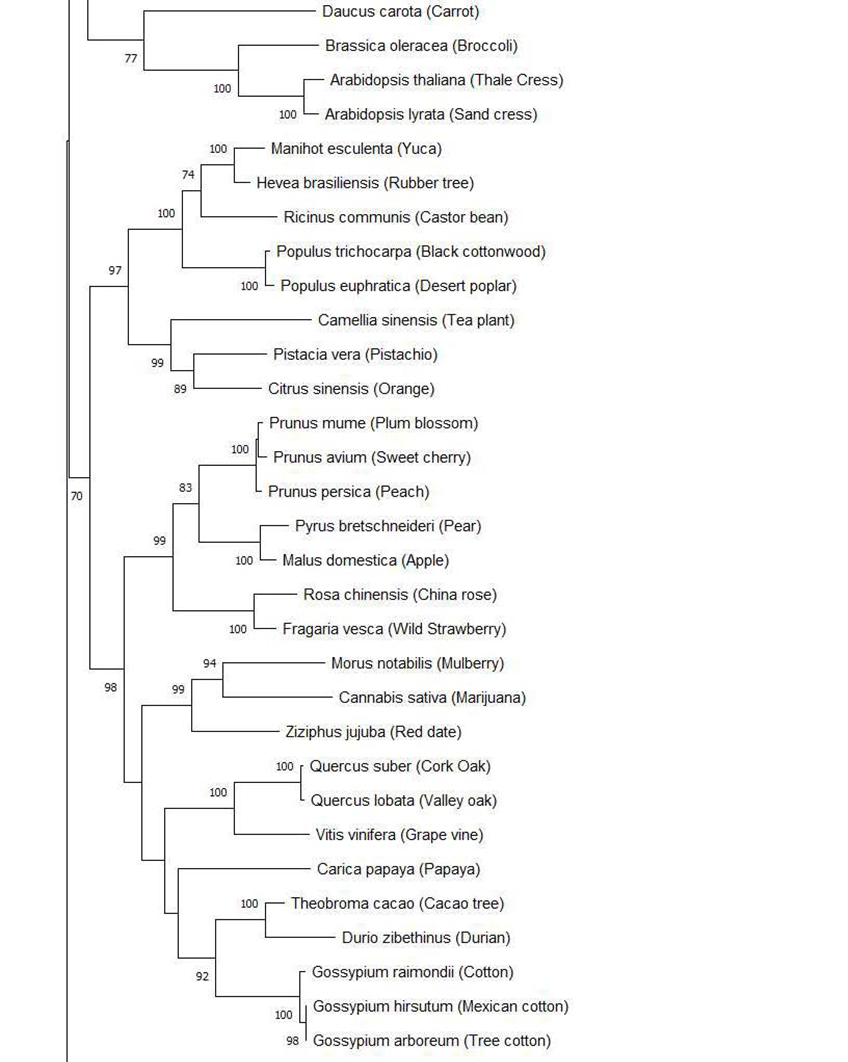
**

**
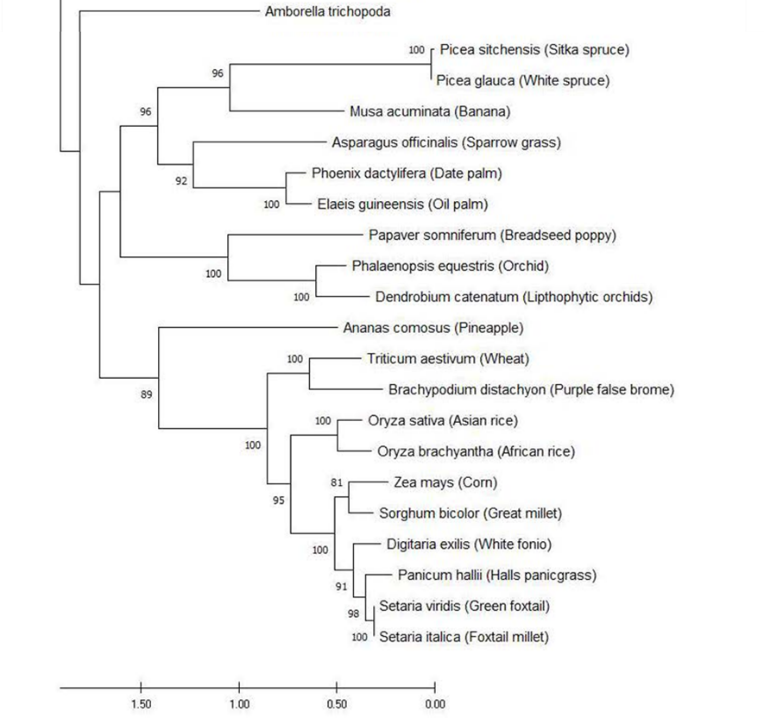
**

**Figure S1.** MEGA-constructed phylogenetic tree based on amino acid sequences of plant KEDs. Detailed description of using MEGA (Molecular Evolutionary Genetics Analysis) for constructing phylogenetic trees is in “Materials and methods”.


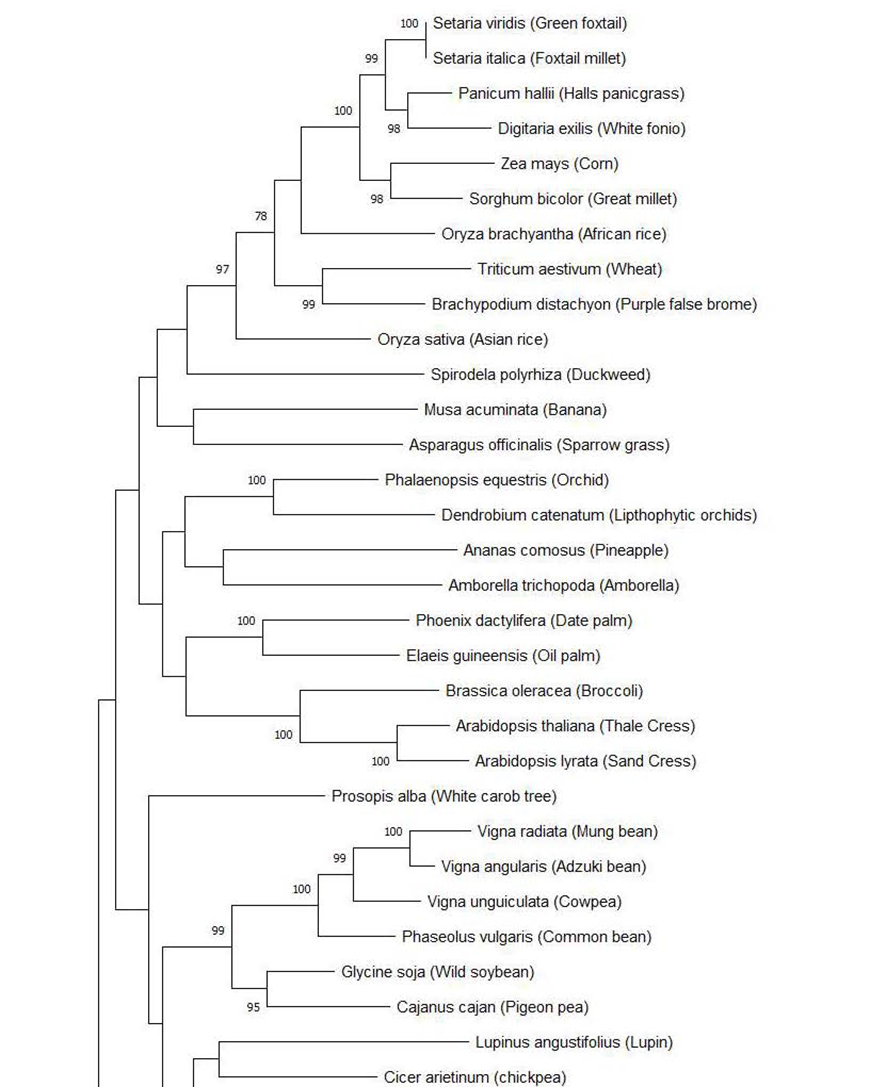


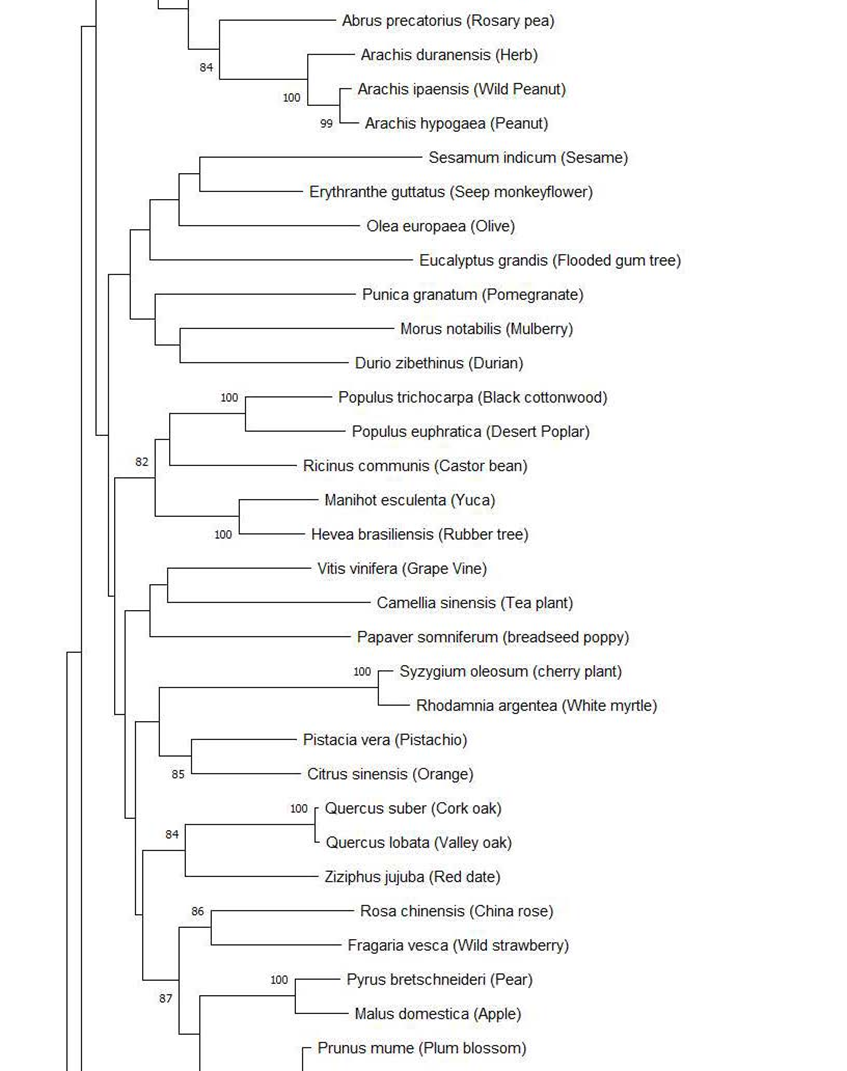


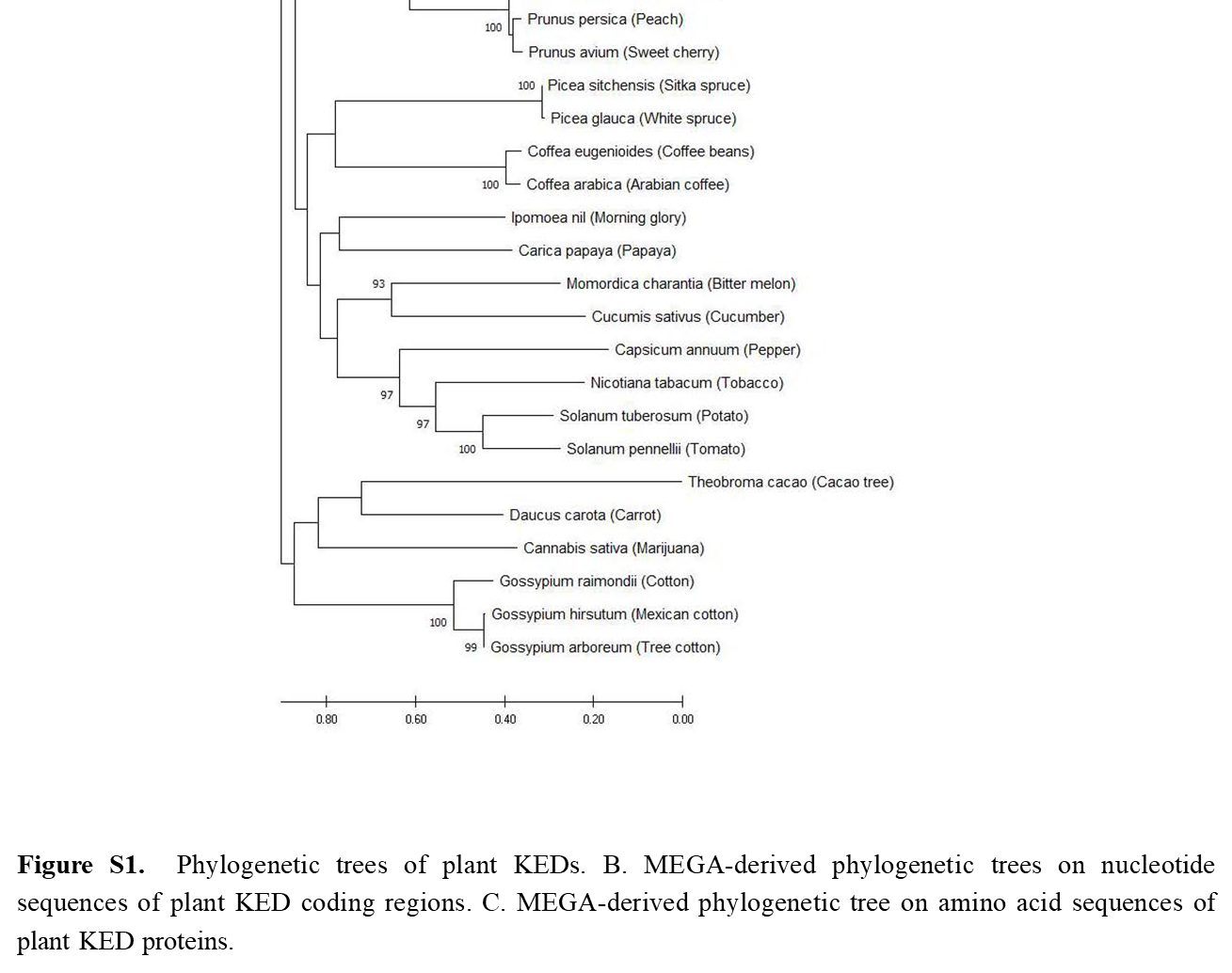


**Figure S2.** MEGA-derived phylogenetic tree based on nucleotide sequences of plant KED coding regions.

**A.**

**
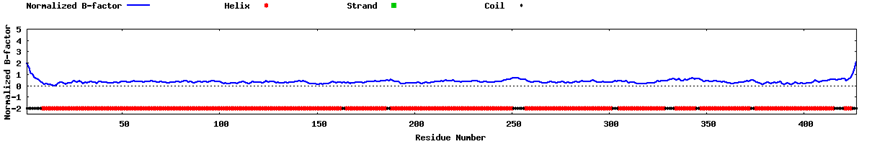
**

**B.**

**
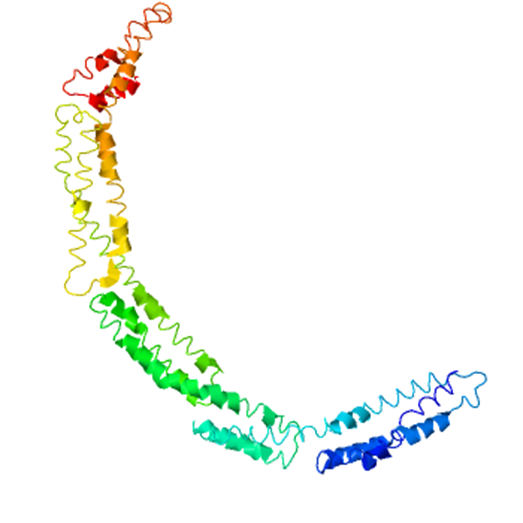
**

**Figure S3**. I-TASSER computer simulation analysis of tomato KED amino acid sequence. A. Normalized B-factor. The lack of negative values indicates very low stability of the tertiary structure. B. Predicted linear, un-structured 3-dimensional conformation, with the N-terminal colored blue and the C-terminal colored red.
